# Supplementary material for: ProteinShader: illustrative rendering of macromolecules
Source: BMC Struct Biol. 2009 Mar 30;9:19. doi: 10.1186/1472-6807-9-19 (PMC2672931; doi:10.1186/1472-6807-9-19)
Supplement: Additional file 1 — ProteinShader program without source code. This compressed file contains the complete ProteinShader program including associated libraries, but no source code. A README.txt file gives an overview of the ProteinShader distribution, and the index.html file in the help subdirectory has directions on getting started with the program as well as a set of tutorials. [file 1472-6807-9-19-S1.zip › ProteinShader-beta-0_9_4-binary/help/api/org/proteinshader/gui/components/controlpanel/VisibilityPanel.html]

VisibilityPanel (ProteinShader API)


|  |  |  |
| --- | --- | --- |
| **Overview** \| **Package** \| **Class** \| **Use** \| **Tree** \| **Deprecated** \| **Index** \| **Help** | |  |
| **PREV CLASS**   NEXT CLASS | **FRAMES**    **NO FRAMES**     **All Classes** |  |
| SUMMARY: NESTED \| FIELD \| CONSTR \| METHOD | DETAIL: FIELD \| CONSTR \| METHOD |  |


---


## org.proteinshader.gui.components.controlpanel Class VisibilityPanel

```
java.lang.Object
  java.awt.Component
      java.awt.Container
          javax.swing.JComponent
              javax.swing.JPanel
                  org.proteinshader.gui.components.controlpanel.VisibilityPanel
```

**All Implemented Interfaces:**: ImageObserver, MenuContainer, Serializable, Accessible

**Direct Known Subclasses:**: AtomVisibilityPanel, CartoonVisibilityPanel

---

``` public abstract class VisibilityPanel extends JPanel ```

This control panel allows the user to modify the visibility status and
alpha value of Drawable objects.

**See Also:**: Serialized Form

---

**Nested Class Summary**

**Nested classes/interfaces inherited from class javax.swing.JPanel**

`JPanel.AccessibleJPanel`

**Nested classes/interfaces inherited from class javax.swing.JComponent**

`JComponent.AccessibleJComponent`

**Nested classes/interfaces inherited from class java.awt.Container**

`Container.AccessibleAWTContainer`

**Nested classes/interfaces inherited from class java.awt.Component**

`Component.AccessibleAWTComponent, Component.BltBufferStrategy, Component.FlipBufferStrategy`


| **Field Summary** | |
| --- | --- |
| `static int` | `SLIDER_INIT`             The initial value for the slider is 75 %. |

**Fields inherited from class javax.swing.JComponent**

`accessibleContext, listenerList, TOOL_TIP_TEXT_KEY, ui, UNDEFINED_CONDITION, WHEN_ANCESTOR_OF_FOCUSED_COMPONENT, WHEN_FOCUSED, WHEN_IN_FOCUSED_WINDOW`

**Fields inherited from class java.awt.Component**

`BOTTOM_ALIGNMENT, CENTER_ALIGNMENT, LEFT_ALIGNMENT, RIGHT_ALIGNMENT, TOP_ALIGNMENT`

**Fields inherited from interface java.awt.image.ImageObserver**

`ABORT, ALLBITS, ERROR, FRAMEBITS, HEIGHT, PROPERTIES, SOMEBITS, WIDTH`


**Constructor Summary**

`VisibilityPanel(Mediator mediator,
Frame dialogOwner,
RadioPanel radioPanel)`
  
          Constructs a VisibilityPanel.


| **Method Summary** | |
| --- | --- |
| `void` | `applyVisibility(VisibilityEnum visibility, float alpha)`             Applies the requested visiblity status and alpha value to whatever item or items are currently selected in the RadioPanel. |
| `float` | `getPercentField()`             Returns the number entered in the "% Translucent" text field. |
| `int` | `getSliderValue()`             Gets the current value for the slider. |
| `void` | `modifyGlobal(AtomModifier atomModifier, BondModifier bondModifier, SegmentModifier segmentModifier)`             Passes the DrawableModifiers to the modifyGlobal() method of the RadioPanel. |
| `abstract  void` | `modifyGlobal(VisibilityEnum visibility, float alpha)`             This helper method for applyVisibility() sets up whatever DrawableModifiers are needed and then calls on the modifyGlobal() method of the radio panel. |
| `void` | `modifyHelices(AtomModifier atomModifier, BondModifier bondModifier, SegmentModifier segmentModifier)`             Passes the DrawableModifiers to the modifyHelices() method of the RadioPanel. |
| `abstract  void` | `modifyHelices(VisibilityEnum visibility, float alpha)`             This helper method for applyVisibility() sets up whatever DrawableModifiers are needed and then calls on the modifyHelices() method of the radio panel. |
| `void` | `modifyLoops(AtomModifier atomModifier, BondModifier bondModifier, SegmentModifier segmentModifier)`             Passes the DrawableModifiers to the modifyLoops() method of the RadioPanel. |
| `abstract  void` | `modifyLoops(VisibilityEnum visibility, float alpha)`             This helper method for applyVisibility() sets up whatever DrawableModifiers are needed and then calls on the modifyLoops() method of the radio panel. |
| `void` | `modifySelected(AtomModifier atomModifier, BondModifier bondModifier, SegmentModifier segmentModifier)`             Passes the DrawableModifiers to the modifySelected() method of the RadioPanel. |
| `abstract  void` | `modifySelected(VisibilityEnum visibility, float alpha)`             This helper method for applyVisibility() sets up whatever DrawableModifiers are needed and then calls on the modifySelected() method of the radio panel. |
| `void` | `modifyStrands(AtomModifier atomModifier, BondModifier bondModifier, SegmentModifier segmentModifier)`             Passes the DrawableModifiers to the modifyStrands() method of the RadioPanel. |
| `abstract  void` | `modifyStrands(VisibilityEnum visibility, float alpha)`             This helper method for applyVisibility() sets up whatever DrawableModifiers are needed and then calls on the modifyStrands() method of the radio panel. |
| `void` | `setPercentField(float n)`             Sets the value for the "% Translucent" text field. |
| `void` | `setSliderValue(int n)`             Sets the current value for the slider. |

**Methods inherited from class javax.swing.JPanel**

`getAccessibleContext, getUI, getUIClassID, paramString, setUI, updateUI`

**Methods inherited from class javax.swing.JComponent**

`addAncestorListener, addNotify, addVetoableChangeListener, computeVisibleRect, contains, createToolTip, disable, enable, firePropertyChange, firePropertyChange, firePropertyChange, fireVetoableChange, getActionForKeyStroke, getActionMap, getAlignmentX, getAlignmentY, getAncestorListeners, getAutoscrolls, getBorder, getBounds, getClientProperty, getComponentGraphics, getComponentPopupMenu, getConditionForKeyStroke, getDebugGraphicsOptions, getDefaultLocale, getFontMetrics, getGraphics, getHeight, getInheritsPopupMenu, getInputMap, getInputMap, getInputVerifier, getInsets, getInsets, getListeners, getLocation, getMaximumSize, getMinimumSize, getNextFocusableComponent, getPopupLocation, getPreferredSize, getRegisteredKeyStrokes, getRootPane, getSize, getToolTipLocation, getToolTipText, getToolTipText, getTopLevelAncestor, getTransferHandler, getVerifyInputWhenFocusTarget, getVetoableChangeListeners, getVisibleRect, getWidth, getX, getY, grabFocus, isDoubleBuffered, isLightweightComponent, isManagingFocus, isOpaque, isOptimizedDrawingEnabled, isPaintingTile, isRequestFocusEnabled, isValidateRoot, paint, paintBorder, paintChildren, paintComponent, paintImmediately, paintImmediately, print, printAll, printBorder, printChildren, printComponent, processComponentKeyEvent, processKeyBinding, processKeyEvent, processMouseEvent, processMouseMotionEvent, putClientProperty, registerKeyboardAction, registerKeyboardAction, removeAncestorListener, removeNotify, removeVetoableChangeListener, repaint, repaint, requestDefaultFocus, requestFocus, requestFocus, requestFocusInWindow, requestFocusInWindow, resetKeyboardActions, reshape, revalidate, scrollRectToVisible, setActionMap, setAlignmentX, setAlignmentY, setAutoscrolls, setBackground, setBorder, setComponentPopupMenu, setDebugGraphicsOptions, setDefaultLocale, setDoubleBuffered, setEnabled, setFocusTraversalKeys, setFont, setForeground, setInheritsPopupMenu, setInputMap, setInputVerifier, setMaximumSize, setMinimumSize, setNextFocusableComponent, setOpaque, setPreferredSize, setRequestFocusEnabled, setToolTipText, setTransferHandler, setUI, setVerifyInputWhenFocusTarget, setVisible, unregisterKeyboardAction, update`

**Methods inherited from class java.awt.Container**

`add, add, add, add, add, addContainerListener, addImpl, addPropertyChangeListener, addPropertyChangeListener, applyComponentOrientation, areFocusTraversalKeysSet, countComponents, deliverEvent, doLayout, findComponentAt, findComponentAt, getComponent, getComponentAt, getComponentAt, getComponentCount, getComponents, getComponentZOrder, getContainerListeners, getFocusTraversalKeys, getFocusTraversalPolicy, getLayout, getMousePosition, insets, invalidate, isAncestorOf, isFocusCycleRoot, isFocusCycleRoot, isFocusTraversalPolicyProvider, isFocusTraversalPolicySet, layout, list, list, locate, minimumSize, paintComponents, preferredSize, printComponents, processContainerEvent, processEvent, remove, remove, removeAll, removeContainerListener, setComponentZOrder, setFocusCycleRoot, setFocusTraversalPolicy, setFocusTraversalPolicyProvider, setLayout, transferFocusBackward, transferFocusDownCycle, validate, validateTree`

**Methods inherited from class java.awt.Component**

`action, add, addComponentListener, addFocusListener, addHierarchyBoundsListener, addHierarchyListener, addInputMethodListener, addKeyListener, addMouseListener, addMouseMotionListener, addMouseWheelListener, bounds, checkImage, checkImage, coalesceEvents, contains, createImage, createImage, createVolatileImage, createVolatileImage, disableEvents, dispatchEvent, enable, enableEvents, enableInputMethods, firePropertyChange, firePropertyChange, firePropertyChange, firePropertyChange, firePropertyChange, firePropertyChange, getBackground, getBounds, getColorModel, getComponentListeners, getComponentOrientation, getCursor, getDropTarget, getFocusCycleRootAncestor, getFocusListeners, getFocusTraversalKeysEnabled, getFont, getForeground, getGraphicsConfiguration, getHierarchyBoundsListeners, getHierarchyListeners, getIgnoreRepaint, getInputContext, getInputMethodListeners, getInputMethodRequests, getKeyListeners, getLocale, getLocation, getLocationOnScreen, getMouseListeners, getMouseMotionListeners, getMousePosition, getMouseWheelListeners, getName, getParent, getPeer, getPropertyChangeListeners, getPropertyChangeListeners, getSize, getToolkit, getTreeLock, gotFocus, handleEvent, hasFocus, hide, imageUpdate, inside, isBackgroundSet, isCursorSet, isDisplayable, isEnabled, isFocusable, isFocusOwner, isFocusTraversable, isFontSet, isForegroundSet, isLightweight, isMaximumSizeSet, isMinimumSizeSet, isPreferredSizeSet, isShowing, isValid, isVisible, keyDown, keyUp, list, list, list, location, lostFocus, mouseDown, mouseDrag, mouseEnter, mouseExit, mouseMove, mouseUp, move, nextFocus, paintAll, postEvent, prepareImage, prepareImage, processComponentEvent, processFocusEvent, processHierarchyBoundsEvent, processHierarchyEvent, processInputMethodEvent, processMouseWheelEvent, remove, removeComponentListener, removeFocusListener, removeHierarchyBoundsListener, removeHierarchyListener, removeInputMethodListener, removeKeyListener, removeMouseListener, removeMouseMotionListener, removeMouseWheelListener, removePropertyChangeListener, removePropertyChangeListener, repaint, repaint, repaint, resize, resize, setBounds, setBounds, setComponentOrientation, setCursor, setDropTarget, setFocusable, setFocusTraversalKeysEnabled, setIgnoreRepaint, setLocale, setLocation, setLocation, setName, setSize, setSize, show, show, size, toString, transferFocus, transferFocusUpCycle`

**Methods inherited from class java.lang.Object**

`clone, equals, finalize, getClass, hashCode, notify, notifyAll, wait, wait, wait`

**Field Detail**

### SLIDER\_INIT

```
public static final int SLIDER_INIT
```

:   The initial value for the slider is 75 %.

    **See Also:**: Constant Field Values


**Constructor Detail**

### VisibilityPanel

```
public VisibilityPanel(Mediator mediator,
                       Frame dialogOwner,
                       RadioPanel radioPanel)
```

:   Constructs a VisibilityPanel.

    **Parameters:**: `mediator` - the centralized Mediator that most listeners need to call on to accomplish their task.: `dialogOwner` - the owner of any Dialogs opened from the SelectorPanel or null if there is no owner).: `radioPanel` - the common RadioPanel used by subpanels.


**Method Detail**

### applyVisibility

```
public void applyVisibility(VisibilityEnum visibility,
                            float alpha)
```

:   Applies the requested visiblity status and alpha value to whatever
    item or items are currently selected in the RadioPanel. The
    VisibilityEnum is OPAQUE, INVISIBLE, and TRANSLUCENT. If an alpha
    value is less than 0.0 or greater than 1.0, an option pane will be
    used to inform the user that there is an out of range error.

    :   **Parameters:**: `visibility` - the visibility status as a VisibilityEnum.: `alpha` - the alpha value associated with the RGB color.

---


### getSliderValue

```
public int getSliderValue()
```

:   Gets the current value for the slider.

    :   **Returns:**: The int value the slider is set to.

---


### setSliderValue

```
public void setSliderValue(int n)
```

:   Sets the current value for the slider. Using this method to
    update the slider will cause any listeners for the slider to be
    called. The value must be between 0 and 100, inclusive, or it
    will be ignored.

    :   **Parameters:**: `n` - the int value to set the slider to.

---


### getPercentField

```
public float getPercentField()
```

:   Returns the number entered in the "% Translucent" text field.

    :   **Returns:**: The translucence value as a float.

---


### setPercentField

```
public void setPercentField(float n)
```

:   Sets the value for the "% Translucent" text field.

    :   **Parameters:**: `n` - the float value to set the text field value to.

---


### modifySelected

```
public abstract void modifySelected(VisibilityEnum visibility,
                                    float alpha)
                             throws ColorOutOfRangeException
```

:   This helper method for applyVisibility() sets up whatever
    DrawableModifiers are needed and then calls on the
    modifySelected() method of the radio panel.

    :   **Parameters:**: `visibility` - the visibility state.: `alpha` - the alpha value associated with the RGB color. **Throws:**: `ColorOutOfRangeException` - if alpha is less than 0.0 or greater than 1.0.

---


### modifyHelices

```
public abstract void modifyHelices(VisibilityEnum visibility,
                                   float alpha)
                            throws ColorOutOfRangeException
```

:   This helper method for applyVisibility() sets up whatever
    DrawableModifiers are needed and then calls on the modifyHelices()
    method of the radio panel.

    :   **Parameters:**: `visibility` - the visibility state.: `alpha` - the alpha value associated with the RGB color. **Throws:**: `ColorOutOfRangeException` - if alpha is less than 0.0 or greater than 1.0.

---


### modifyStrands

```
public abstract void modifyStrands(VisibilityEnum visibility,
                                   float alpha)
                            throws ColorOutOfRangeException
```

:   This helper method for applyVisibility() sets up whatever
    DrawableModifiers are needed and then calls on the modifyStrands()
    method of the radio panel.

    :   **Parameters:**: `visibility` - the visibility state.: `alpha` - the alpha value associated with the RGB color. **Throws:**: `ColorOutOfRangeException` - if alpha is less than 0.0 or greater than 1.0.

---


### modifyLoops

```
public abstract void modifyLoops(VisibilityEnum visibility,
                                 float alpha)
                          throws ColorOutOfRangeException
```

:   This helper method for applyVisibility() sets up whatever
    DrawableModifiers are needed and then calls on the modifyLoops()
    method of the radio panel.

    :   **Parameters:**: `visibility` - the visibility state.: `alpha` - the alpha value associated with the RGB color. **Throws:**: `ColorOutOfRangeException` - if alpha is less than 0.0 or greater than 1.0.

---


### modifyGlobal

```
public abstract void modifyGlobal(VisibilityEnum visibility,
                                  float alpha)
                           throws ColorOutOfRangeException
```

:   This helper method for applyVisibility() sets up whatever
    DrawableModifiers are needed and then calls on the modifyGlobal()
    method of the radio panel.

    :   **Parameters:**: `visibility` - the visibility state.: `alpha` - the alpha value associated with the RGB color. **Throws:**: `ColorOutOfRangeException` - if alpha is less than 0.0 or greater than 1.0.

---


### modifySelected

```
public void modifySelected(AtomModifier atomModifier,
                           BondModifier bondModifier,
                           SegmentModifier segmentModifier)
```

:   Passes the DrawableModifiers to the modifySelected() method of the
    RadioPanel.

    :   **Parameters:**: `atomModifier` - an AtomModifier programmed to modify Atoms (or null for no modifications).: `bondModifier` - a BondModifier programmed to modify Bonds (or null for no modifications).: `segmentModifier` - a SegmentModifier programmed to modify Segments (or null for no modifications).

---


### modifyHelices

```
public void modifyHelices(AtomModifier atomModifier,
                          BondModifier bondModifier,
                          SegmentModifier segmentModifier)
```

:   Passes the DrawableModifiers to the modifyHelices() method of the
    RadioPanel.

    :   **Parameters:**: `atomModifier` - an AtomModifier programmed to modify Atoms (or null for no modifications).: `bondModifier` - a BondModifier programmed to modify Bonds (or null for no modifications).: `segmentModifier` - a SegmentModifier programmed to modify Segments (or null for no modifications).

---


### modifyStrands

```
public void modifyStrands(AtomModifier atomModifier,
                          BondModifier bondModifier,
                          SegmentModifier segmentModifier)
```

:   Passes the DrawableModifiers to the modifyStrands() method of the
    RadioPanel.

    :   **Parameters:**: `atomModifier` - an AtomModifier programmed to modify Atoms (or null for no modifications).: `bondModifier` - a BondModifier programmed to modify Bonds (or null for no modifications).: `segmentModifier` - a SegmentModifier programmed to modify Segments (or null for no modifications).

---


### modifyLoops

```
public void modifyLoops(AtomModifier atomModifier,
                        BondModifier bondModifier,
                        SegmentModifier segmentModifier)
```

:   Passes the DrawableModifiers to the modifyLoops() method of the
    RadioPanel.

    :   **Parameters:**: `atomModifier` - an AtomModifier programmed to modify Atoms (or null for no modifications).: `bondModifier` - a BondModifier programmed to modify Bonds (or null for no modifications).: `segmentModifier` - a SegmentModifier programmed to modify Segments (or null for no modifications).

---


### modifyGlobal

```
public void modifyGlobal(AtomModifier atomModifier,
                         BondModifier bondModifier,
                         SegmentModifier segmentModifier)
```

:   Passes the DrawableModifiers to the modifyGlobal() method of the
    RadioPanel.

    :   **Parameters:**: `atomModifier` - an AtomModifier programmed to modify Atoms (or null for no modifications).: `bondModifier` - a BondModifier programmed to modify Bonds (or null for no modifications).: `segmentModifier` - a SegmentModifier programmed to modify Segments (or null for no modifications).


---


|  |  |  |
| --- | --- | --- |
| **Overview** \| **Package** \| **Class** \| **Use** \| **Tree** \| **Deprecated** \| **Index** \| **Help** | |  |
| **PREV CLASS**   NEXT CLASS | **FRAMES**    **NO FRAMES**     **All Classes** |  |
| SUMMARY: NESTED \| FIELD \| CONSTR \| METHOD | DETAIL: FIELD \| CONSTR \| METHOD |  |


---

# *Copyright © 2007-2008*
